# A Comparison of Manual versus Electric Bicycle Injuries Presenting to a Pediatric Emergency Department

**DOI:** 10.5041/RMMJ.10370

**Published:** 2019-07-18

**Authors:** Tali Capua, Miguel Glatstein, Karin Hermon, Oren Tavor, Dennis Scolnik, Veronika Kusaev, Ayelet Rimon

**Affiliations:** 1 Pediatric Emergency Medicine, Dana-Dwek Children’s Hospital, Sackler School of Medicine, Tel Aviv University, Tel Aviv, Israel; 2 Division of Clinical Pharmacology and Toxicology, Tel Aviv Sourasky Medical Center, Tel Aviv University, Tel Aviv, Israel; 3 Divisions of Pediatric Emergency Medicine and Clinical Pharmacology and Toxicology, Department of Pediatrics, The Hospital for Sick Children, University of Toronto, Ontario, Canada

**Keywords:** Electric bicycles, injuries, pediatrics, Tel Aviv

## Abstract

**Background:**

The use of electric bicycles (E-bikes) has dramatically increased over the last decade. E-bikes offer an inexpensive, alternative form of transport, but also pose a new public health challenge in terms of safety and injury prevention.

**Objective:**

The aim of this study was to describe the epidemiology and severity of E-bike related injuries among children treated in the emergency department (ED) and to compare these to manual bicycle related injuries.

**Methods:**

A retrospective observational study of all pediatric patients presenting to the ED between December 2014 and November 2015 with an injury related to E-bike or manual bicycle use. Data including demographics, diagnosis, injury severity score (ISS), and outcome were compared.

**Results:**

A total of 196 cyclist injuries presented to the ED; 85 related to E-bike use and 111 to manual bicycle riders. The mean age of E-bikers was 13.7 years (7.5–16 years) and of manual bicycle riders was 9.9 years (3–16 years). Injuries to the head and the extremities were common in both groups. E-bikers had significantly more intra-abdominal organ injury (*P*=0.047). Injury severity scores were low overall, but injuries of higher severity (ISS>9) only occurred among the E-bikers.

**Conclusions:**

Pediatric E-bike injuries tend to be more severe than those sustained during manual bicycle riding. Further research into bicycle and other road and pavement users could lead to enhanced regulation regarding E-bike usage.

## INTRODUCTION

One of the leading causes of non-fatal injury among children is bicycle-riding related injuries. A popular recreation for people of all ages is bike riding, with related injuries causing significant morbidity and mortality.^1^ Children are especially at risk given the popularity of bicycle riding within this age group. Electric bikes (E-bikes) and electric scooters are part of a wide range of light electric vehicles (LEVs) that provide convenient local transportation. While E-bikes offer convenient, environmentally friendly, and cheap transport, they pose a new public health challenge in terms of safety and injury prevention.^2^–^4^ The use of E-bikes has dramatically increased over the last decade. By 2016 approximately 466 million E-bikes had been purchased, and the demand for E-bikes is expected to increase.^5^ Local governments promote the use of these LEVs due to their ecological advantage.^6^ Legislation for electric scooters and for E-bikes was introduced in Israel in 2004 and 2009, respectively, and since then sales have soared.

In 2010 a total of 1200 E-bikes were sold in Israel; in 2011 6000 and in 2013 30,000 E-bikes were purchased, representing an annual growth rate of 800%.^7^

Since 2013 Tel Aviv, Israel’s biggest metropolis, has witnessed an alarming rise in media reports regarding the number and severity of E-bike related injuries, both in riders and in pedestrians.^8^ We have previously described E-bike related injuries in children,^7^ but the literature on E-bike related injuries remains sparse. Furthermore, the differences between E-bike and manual bicycle related injuries in children have not yet been investigated. Documenting differences between E-bike and manual bicycle related injuries may influence related legislation and enforcement.

The aim of this study was to describe the incidence, circumstances, and severity of E-bike related injuries among children treated in the pediatric emergency department (PED) in comparison to manual bicycle related injuries.

## MATERIAL AND METHODS

We reviewed the medical charts of all patients, up to the age of 16 years, who presented to our PED following a bicycle related injury, between December 2014 and November 2015. The Tel Aviv Sourasky Medical Center is an urban, tertiary care center, designated a Level One trauma center. Our center sees >28,000 children in the pediatric emergency room per year.

Patients were included if they were the primary rider of either an electric or manual bicycle involved in a collision. Extracted data included age, gender, details of the accident, as well as severity of injury, medical diagnosis, and outcome. Abbreviated injury score (AIS) and injury severity score (ISS) were calculated for each patient.^9^ The study protocol was approved by the institutional ethics committee.

Statistical analysis was performed using IBM SPSS statistics version 24. Categorical variables were described using frequency and percentage, and sequential variables were described using mean and standard deviation. Differences in sequential variables were compared using the Fisher Exact Test, and difference between means was performed using one-sided *t* test for independent means. *P* values <0.05 were considered statistically significant.

## RESULTS

During the 12-month study period a total of 221 patients with bicycle related injuries presented to the PED. Once pedestrians and bicycle passengers were removed from the data, bicycle riders constituted a group of 196 children: 85 E-bikers and 111 manual bicycle riders. The demographic and clinical features of the patients are presented in Table 1. Mean age of E-bike riders was significantly higher than that of manual bicycle riders (13.7 versus 9.9 years, *P*=0.00001), with a similar male predominance. Use of a helmet was under-documented in this study (documented in less than one-third of the medical records), but was significantly less used amongst E-bike riders.

**Table 1:** Clinical Characteristics of Patients Involved in Bicycle Related Collisions.

Clinical Characteristic	E-bike (*n*=85)	Manual Bicycle (*n*=111)	*P* Value
Mean Age, Years (±SD)	13.7 (±2.9)	9.9 (±3.4)	0.00001

Male Gender, %	67	64	0.76

Helmet Use, *n* Positive/*n* Documented (%)	6/32 (19)	13/27 (48)	0.02

Body Region Injured, *n* (%)			
Head	34 (40)	46 (42)	0.88

Neck	7 (8)	3 (3)	0.11

Chest	10 (12)	3 (3)	0.02

Abdomen	15 (18)	16 (14)	0.56

Back	2 (2)	1 (0.9)	0.58

Upper Extremities	34 (40)	42 (38)	0.77

Lower Extremities	35 (41)	28 (25)	0.02

Injury Types, *n* (%)			
Superficial Skin Injury	45 (53)	64 (58)	0.56

Skin Laceration	22 (26)	27 (24)	0.87

Limb Fracture	21 (25)	27 (24)	1.00

Intra-abdominal Organ(s) Injury	7 (8)	2 (2)	0.047

Intracranial Hemorrhage	1 (1)	1 (1)	1.00

Outcome			
Admission, *n* (%)	12 (14)	17 (15)	0.84

Surgery, *n* (%)	4 (4.7)	0 (0)	0.02

ISS, Mean (±SD)	3.1 (±3.2)	2.5 (±0.5)	0.049

ISS, Median (Range)	2 (1–16)	1 (1–9)	

ISS, injury severity score.

Body areas injured were different between the groups, with a higher proportion of chest and lower limb injuries in the E-bike group (*P*=0.02) and more injuries to intra-abdominal organs (*P*=0.047) in the E-bike group. Twelve (14%) E-bike riders and 17 (15%) manual bicycle riders were admitted. Surgical intervention was needed only in the E-bike group (4.7%). The ISS was low overall, with a significantly higher mean ISS in the E-bike group. Injuries of higher severity (ISS>9) were found only among the injured E-bikers.

## DISCUSSION

We previously conducted a study describing injuries related to E-bike riding in a large pediatric population.^7^ That study prompted us to compare E-bike to manual bicycle related injuries. To the best of our knowledge, this is the first study to investigate these differences in a pediatric population. Our data showed that E-bike riders tend to be older (13.7 versus 9.9 years, *P*=0.00001), less consistent in helmet use, and tend to suffer more injuries to the chest, lower limbs, and intra-abdominal organs. Consistent with previous studies, injured bicycle riders were predominantly male.^3^–^5^,^7^,^10^ No cases of mortality occurred, and the average ISS was relatively low, comparable with numbers quoted in adult E-bike studies.^11^

In 2012, when E-bikes were first introduced in Israel, use was common even among young children, but following documentation of E-bike related injuries,^7^ Israel tightened its legislation on E-bike usage. In 2016, Israel passed legislature limiting LEV use to persons aged 16 and older, limiting LEV speed to a maximum of 25 kilometers per hour, and requiring riders to wear a helmet.^12^ In 2019, strict laws were created requiring a special LEV license for those teenagers aged 16–18 without an automobile license.^12^ Our study took place before these laws were put into effect. We expect to see a significant decrease in E-bike injuries in the pediatric population following implementation of these new laws. However, such laws are extremely difficult to implement, despite the effort put into them, and little is known about their potential to reduce injuries.

A study of adult E-bikers in Switzerland described a higher rate of mild head injury even with a high rate (75%) of helmet use.^13^ Although helmet use was not consistently recorded in our study, it did seem to be significantly lower in the E-bike group (19% versus 48%, *P*=0.02), suggesting a dangerous pattern of behavior among E-bikers. This is alarming, considering that there is evidence for the effectiveness of helmets in reducing bicyclist and motorcyclist head injuries.^13^ Routine documentation of helmet use in patients suffering bicycle or motorcycle related injuries would facilitate future research and strengthen policy decisions.

Although mean ISS of all bicycle related injuries was low, higher scores (ISS>9) were found only in E-bikers. This is consistent with previous studies which found that E-bikers suffered more severe injuries and also experienced multiple injuries more frequently.^5^ A significantly higher number of E-bikers experienced injury to intra-abdominal organs (*P*=0.047). This may be due to the faster speeds attained by E-bikes.^14^

## LIMITATIONS

Our study is limited in being retrospective in nature, with the consequence that details of the injury, including helmet use, were not always available. Secondly, our sample size is relatively modest.

## CONCLUSIONS

Our study suggests that E-biking children are more severely injured. Parents and children should be educated about the risks of LEVs, to furnish them with data to evaluate the need to resist the peer pressure encouraging purchase of an E-bike. They should be made aware of the higher possibility of severe injuries associated with E-bike use. Measures to prevent injury, such as riding lessons and helmet use, should be encouraged. Legislation aimed at preventing pediatric E-bike related injuries should be enforced, and new regulations enhancing the safety of those old enough to use E-bikes should be considered. E-bike related injury is a burgeoning phenomenon globally, and more research is needed to inform policy-makers and industry and enhance the safety of bikers and other road and pavement users. Further studies in Israel should be performed once the new regulations are implemented to measure changes in LEV injury severity and demographics in the pediatric population.

## Abbreviations

AIS: abbreviated injury score
E-bike: electric bicycle
ED: emergency department
ISS: injury severity score
LEV: light electric vehicle
PED: pediatric emergency department.

## References

[1] McAdams RJ, Swidarski K, Clark RM, Roberts KJ, Yang J, Mckenzie LB (2018). Bicycle-related injuries among children treated in US emergency departments, 2006–2015. Accid Anal Prev.

[2] Tenenbaum S, Weltsch D, Bariteau JT (2017). Orthopaedic injuries among electric bicycle users. Injury.

[3] Yang J, Hu Y, Du W (2014). Unsafe riding practice among electric bikers in Suzhou, China: an observational study. BMJ Open.

[4] Du W, Yang J, Powis B (2013). Understanding on-road practices of electric bike riders: an observational study in a developed city of China. Accid Anal Prev.

[5] Poos HPAM, Lefarth TL, Harbers JS, Wendt KW, El Moumni M, Reininga IHF (2017). E-bikers are more often seriously injured in bicycle accidents: results from the Groningen bicycle accident database. Ned Tijdschr Geneeskd.

[6] Gojanovic B, Welker J, Iglesias K, Daucourt C, Gremion G (2011). Electric bicycles as a new active transportation modality to promote health. Med Sci Sports Exerc.

[7] Hermon K, Capua T, Glatstein M, Scolnik D, Tavor O, Rimon A (2018). Pediatric electric bicycle injuries: the experience of a large urban tertiary care pediatric hospital. Pediatr Emerg Care.

[8] Siegel-Itzkovich J (2011). Israel amends law forcing adult cyclists to wear helmets. BMJ.

[9] Gennarelli TA, Wodzin E (2006). AIS 2005: a contemporary injury scale. Injury.

[10] Gross I, Weiss DJ, Eliasi E, Bala M, Hashavya S (2018). E-bike-related trauma in children and adults. J Emerg Med.

[11] Papoutsi S, Martinolli L, Braun CT, Exadaktylos AK (2014). E-bike injuries: experience from an urban emergency department – a retrospective study from Switzerland. Emerg Med Int.

[12] Stoler T (2018). Israel to increase regulation on electric bikes and e-scooters. CTECH by Calcalist website.

[13] Jewett A, Beck LF, Taylor C, Baldwin G (2016). Bicycle helmet use among persons 5years and older in the United States, 2012. J Safety Res.

[14] Hu F, Lv D, Zhu J, Fang J (2014). Related risk factors for injury severity of e-bike and bicycle crashes in Hefei. Traffic Inj Prev.

